# A transgenic tool to assess *Anopheles* mating competitiveness in the field

**DOI:** 10.1186/s13071-018-3218-5

**Published:** 2018-12-24

**Authors:** Andrea L. Smidler, Sean N. Scott, Enzo Mameli, W. Robert Shaw, Flaminia Catteruccia

**Affiliations:** Harvard T.H. Chan School of Public Health, Department of Immunology and Infectious Diseases, Boston, MA USA

## Abstract

**Background:**

Malaria parasites, transmitted by the bite of an anopheline mosquito, pose an immense public health burden on many tropical and subtropical regions. The most important malaria vectors in sub-Saharan Africa are mosquitoes of the *Anopheles gambiae* complex including *An. gambiae* (*sensu stricto*). Given the increasing rates of insecticide resistance in these mosquitoes, alternative control strategies based on the release of genetically modified males are being evaluated to stop transmission by these disease vectors. These strategies rely on the mating competitiveness of release males, however currently there is no method to determine male mating success without sacrificing the female. Interestingly, unlike other insects, during mating *An. gambiae* males transfer their male accessory glands (MAGs) seminal secretions as a coagulated mating plug which is deposited in the female atrium.

**Results:**

Here we exploit this male reproductive feature and validate the use of a MAG-specific promoter to fluorescently label the mating plug and visualize the occurrence of insemination *in vivo*. We used the promoter region of the major mating plug protein, Plugin, to control the expression of a Plugin-tdTomato (PluTo) fusion protein, hypothesizing that this fusion protein could be incorporated into the plug for sexual transfer to the female. *Anopheles gambiae* PluTo transgenic males showed strong red fluorescence specifically in the MAGs and with a pattern closely matching endogenous Plugin expression. Moreover, the fusion protein was integrated into the mating plug and transferred to the female atrium during mating where it could be visualized microscopically *in vivo* without sacrificing the female. PluTo males were equally as competitive at mating as wild type males, and females mated to these males did not show any reduction in reproductive fitness.

**Conclusion:**

The validation of the first MAG-specific promoter in transgenic *An. gambiae* facilitates the live detection of successful insemination hours after copulation has occurred. This provides a valuable tool for the assessment of male mating competitiveness not only in laboratory experiments but also in semi-field and field studies aimed at testing the feasibility of releasing genetically modified mosquitoes for disease control.

**Electronic supplementary material:**

The online version of this article (10.1186/s13071-018-3218-5) contains supplementary material, which is available to authorized users.

## Background

Malaria kills approximately 450,000 people every year, mostly children in sub-Saharan Africa, and infects hundreds of millions more [1]. The *Plasmodium* parasites that cause this devastating disease are transmitted to humans exclusively by anopheline mosquitoes. Although control methods based on the use of long-lasting insecticide-treated nets (LLINs) and indoor residual sprays (IRS) have been very effective at reducing the number of malaria cases and deaths in the past 15 years, increasing levels of insecticide resistance in *Anopheles* populations throughout Africa are threatening the use of these strategies [1]. For example, the majority of anophelines across Africa are now resistant to pyrethroids [2], the only insecticide approved for use on LLINs, while resistance rates to carbamates and organophosphates are on the rise [3]. Alarmingly, populations have emerged with resistance to all four classes of insecticides available for malaria control [4, 5], making the development of novel vector control technologies increasingly urgent.

Targeting fertility for insect control has been successful in a variety of species, and a number of strategies aimed at inducing sterility in field populations are currently being developed in mosquitoes. Sterile Insect Technique (SIT), a strategy based on the mass release of irradiated males to sterilize females upon mating [6], has been successfully implemented against populations of the screwworm *Cochliomyia hominivorax* and the Mediterranean fruit fly *Ceratitis capitata* to reduce the economic burden of these important agricultural pests [7–9]. Adapting SIT and other similar sterilizing technologies (such as chemosterilization) to *Anopheles* vectors of human malaria has been challenging, and requires optimization to maximize competitiveness of colonized, mass-reared and sterilized males [10–14]. To circumvent the potential problems linked to sterility by irradiation or chemosterilants, strategies based on genetic manipulation of insect fertility are being considered. Among the most promising strategies, gene drives are in development for *Anopheles* which could spread infertility through natural mosquito populations by positively biasing their own inheritance. The potential of gene drives to induce sterility in field populations has been recently demonstrated in cages of *An. gambiae* [15], where gene drive transgenes targeting female fertility genes spread for a few generations [16]. Regardless of their mode of action, all genetic control strategies require the release of male mosquitoes that have high mating competitiveness and can successfully mate with field females, making studies into male reproductive fitness a critical requisite for successful implementation.

*Anopheles gambiae* are largely monandrous, which means they mate only once in their lifetime. During copulation seminal secretions produced by the male accessory glands (MAGs) are transferred to the female atrium in the form of a coagulated mating plug [17]. The plug is composed of seminal secretions including proteins [18] and the steroid hormone 20-hydroxyecdysone (20E) [19], and is digested by the female over a period of 24 h following copulation [20]. The loss of female receptivity to further mating is at least partially due to the transfer and function of the steroid hormone 20E [21–23], which also triggers oviposition in blood-fed females and affects other important aspects of the female post-mating physiology, including egg development and fertility [21, 22, 24]. One of the most abundant mating plug proteins is Plugin, a MAG-specific glutamine-rich structural protein that becomes incorporated into the plug upon cross-linking to itself and other seminal proteins by the action of the plug-forming transglutaminase AgTG3 [20, 25]. Transcriptional silencing of *AgTG3* partially prevents plug formation and transfer, which results in severe sperm storage defects in the female, causing infertility [20]. Other less characterized mating plug factors that may play a role in female post-mating physiology include short accessory gland proteins (Acps), a number of proteases and peptidases [26–28], serine protease inhibitors which play a role in mammalian fertility [29], and cysteine-rich secretory folding proteins (CRISPs) that are involved in gamete interactions [30]. While many of these proteins are characterized in other organisms, their role in male mating fitness has not yet been elucidated in *An. gambiae,* making development of novel MAG-specific tools important for addressing these outstanding questions. Studies on the evolutionary trajectory of mating plug formation have shown that transfer of a mating plug is a derived trait not limited to *An. gambiae* but widely conserved across anopheline species from Africa and Southeast Asia, while being absent in males of Central American species like *An. albimanus* [23].

The presence of a mating plug within the female atrium following mating is considered a *de facto* marker of successful copulation given its relevance for sperm storage [20, 21, 31]. To facilitate measuring male mating success *in vivo,* we developed a novel transgenic line, Plugin-tdTomato (PluTo), for use as a marker for the occurrence of insemination in females. PluTo males express a Plugin-tdTomato fusion protein specifically in their MAGs via the *Plugin* promoter, without negatively affecting endogenous Plugin levels. We show that Plugin-tdTomato is incorporated into the mating plug and is transferred to the female during mating, where it is detectable by microscopic examination after copulation. Importantly, mating experiments in competition with wild type males suggest that male mating competitiveness is not affected by expression of this transgene. Moreover, transfer of this fusion protein does not affect the reproductive fitness of females mated to PluTo males. These data demonstrate that this transgenic construct could be useful for determining the mating competitiveness of *An. gambiae* strains, as well as of other anopheline species that transfer a mating plug, in semi-field and field conditions.

## Results

### Generation and characterization of the Plugin-tdTomato transgenic line

**Fig. 1:**
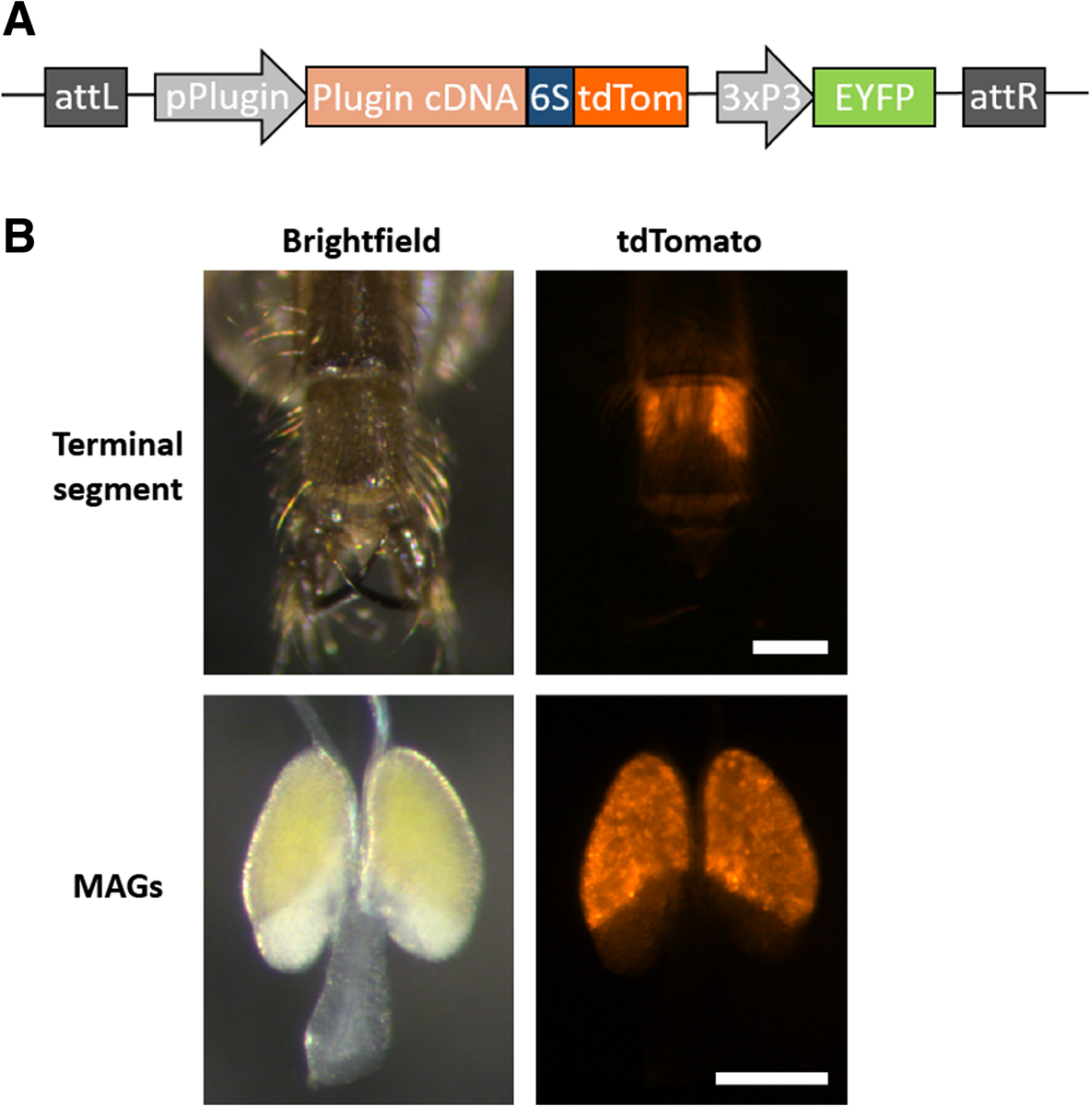
PluTo transgene design and male phenotype. **a** The PluTo transgene is composed of 2688 bp of the 5' regulatory region immediately upstream of the start codon of AGAP009368 followed by Plugin cDNA fused to a 6-serine linker followed by tdTomato. The transgene also carries a 3xP3-EYFP selectable marker cassette for identification of transgenic larvae. Each coding sequence has an SV40 terminator to prevent transcriptional read-through. Following insertion, the flanking recombination sites attL and attR are generated in the genome. **b** Adult PluTo transgenic males display strong tdTomato fluorescence in their MAGs (lower panels). The fluorescence is clearly and easily identifiable under a fluorescent dissecting microscope through the adult male cuticle (upper panels). *Scale-bar*: 100 μm

Plugin is specifically and abundantly expressed within the MAGs [20, 32]. During mating, this seminal protein is transferred to females as part of the mating plug, a gelatinous structure that is composed of multiple other proteins as well as the steroid hormone 20E [20]. To create a transgenic line exhibiting robust MAG-specific expression, we cloned a region comprising 2688 bp upstream of the *Plugin* start codon, likely encompassing the *Plugin* promoter. We then used this region to drive expression of *tdTomato* [33] fused to a *Plugin* cDNA construct [20] to achieve incorporation into the mating plug for transfer to females. The two protein-coding regions of the fusion construct were connected by a 6-serine residue linker (reviewed in [34]) (Fig 1a). The transgenic cassette was cloned into the pDSAY transgenesis plasmid [35] and was injected into *An. gambiae* embryos from the X1 docking line which contains an attP site for φC31-mediated integration on chromosome 2L [35], in conjunction with an integrase-expressing helper plasmid [35]. A single transgenic line was generated and named **Plu**gin-**To**mato (PluTo). Transgene insertion into the X1 docking site was confirmed by PCR (Additional file 1: Figure S1).

This transgenic line displayed strong tdTomato fluorescence in the MAGs, which was visible through the male cuticle beginning at the early adult stage (Fig. 1b). No discernable tdTomato fluorescence was observed in other male or female tissues. In agreement with our microscopic analysis, qRT-PCR demonstrated that the *Plugin* promoter was capable of robust *Plugin*-*tdTomato* expression in the MAGs comparable to endogenous *Plugin* expression, with negligible transcripts observed in the male rest-of-body and female whole-body tissues (Additional file 2: Figure S2a). Moreover, both endogenous Plugin and Plugin-tdTomato fusion proteins were observed exclusively in the MAGs at ~80kDA and ~150kDA respectively, using antibodies targeting Plugin and tdTomato (Additional file 2: Figure S2b, bands 2 and 4 respectively) [20]. Several tdTomato-specific bands were observed that were not recognized by α-Plugin, suggesting the occurrence of cleavage of the fusion protein or use of an alternative start codon after the antibody binding site (Additional file 2: Figure S2b, band 3).

### Plugin-tdTomato is localized to vesicles within the anterior compartment of the MAGs

**Fig. 2:**
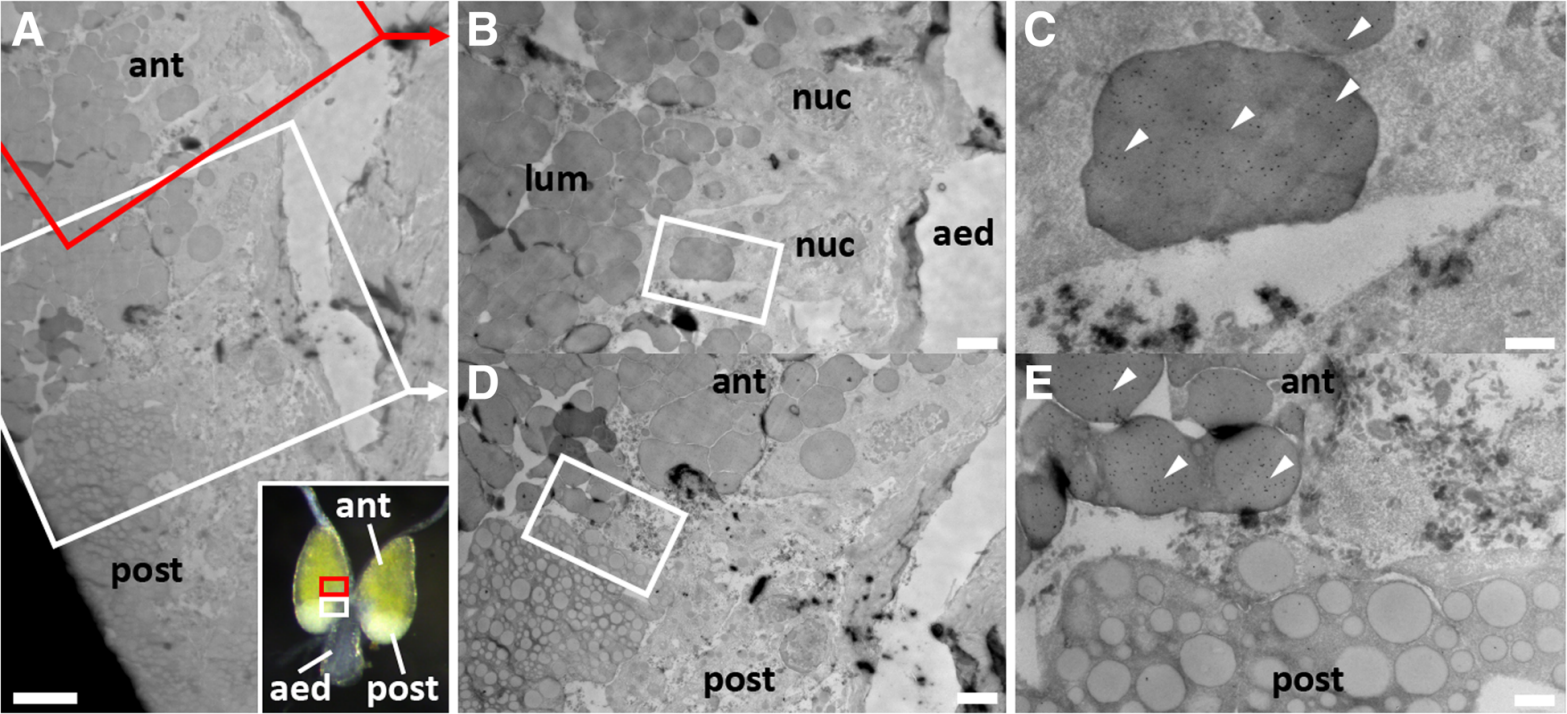
Endogenous Plugin localization in the MAGs. **a** Transmission electron micrograph of the boundary between anterior (ant) and posterior (post) MAG compartments in wild type males. Red box indicates area magnified in panel (**b**), white box indicates area magnified in panel (**d**). Inset image provided for orientation within MAGs and approximate area magnified. **b** The anterior compartment has peripherally located nuclei (nuc) and contains electron-dense vesicles that fill the lumen (lum). The central aedeagus (aed) is marked for orientation. White box indicates area magnified in (**c**). **c** Plugin is localized within the electron-dense secretory vesicles by gold-labelled α-Plugin (white arrowheads). **d** Vesicles within the anterior (ant) and posterior (post) compartments are different in size and electron density, suggesting different contents. White box indicates area magnified in (**e**). **e** Plugin is localized only within the more electron-dense secretory vesicles of the anterior compartment by gold-labelled α-Plugin (white arrowheads). *Scale-bars*: (**a**), 5 μm; (**b**,** d**), 2 μm; (**c**, **e**), 0.5 μm

To determine the expression of Plugin-tdTomato in more detail and compare it to endogenous Plugin, we first characterized the localization of Plugin in the MAGs of wild type mosquitoes by cryo-immune electron microscopy (IEM) using an α-Plugin antibody [20]. In *Anopheles*, the MAGs are composed of a thin epithelial sheath encasing two secretory compartments, a large anterior compartment and a smaller posterior compartment, each containing a single layer of holocrine secretory cells that produce MAG substances of different electron densities [36]. This global organization was observed in our IEM analysis (Fig. 2a), in which we detected multiple Plugin-positive electron-dense vesicles in cells of the anterior compartment only (Fig. 2b-e).

**Fig. 3:**
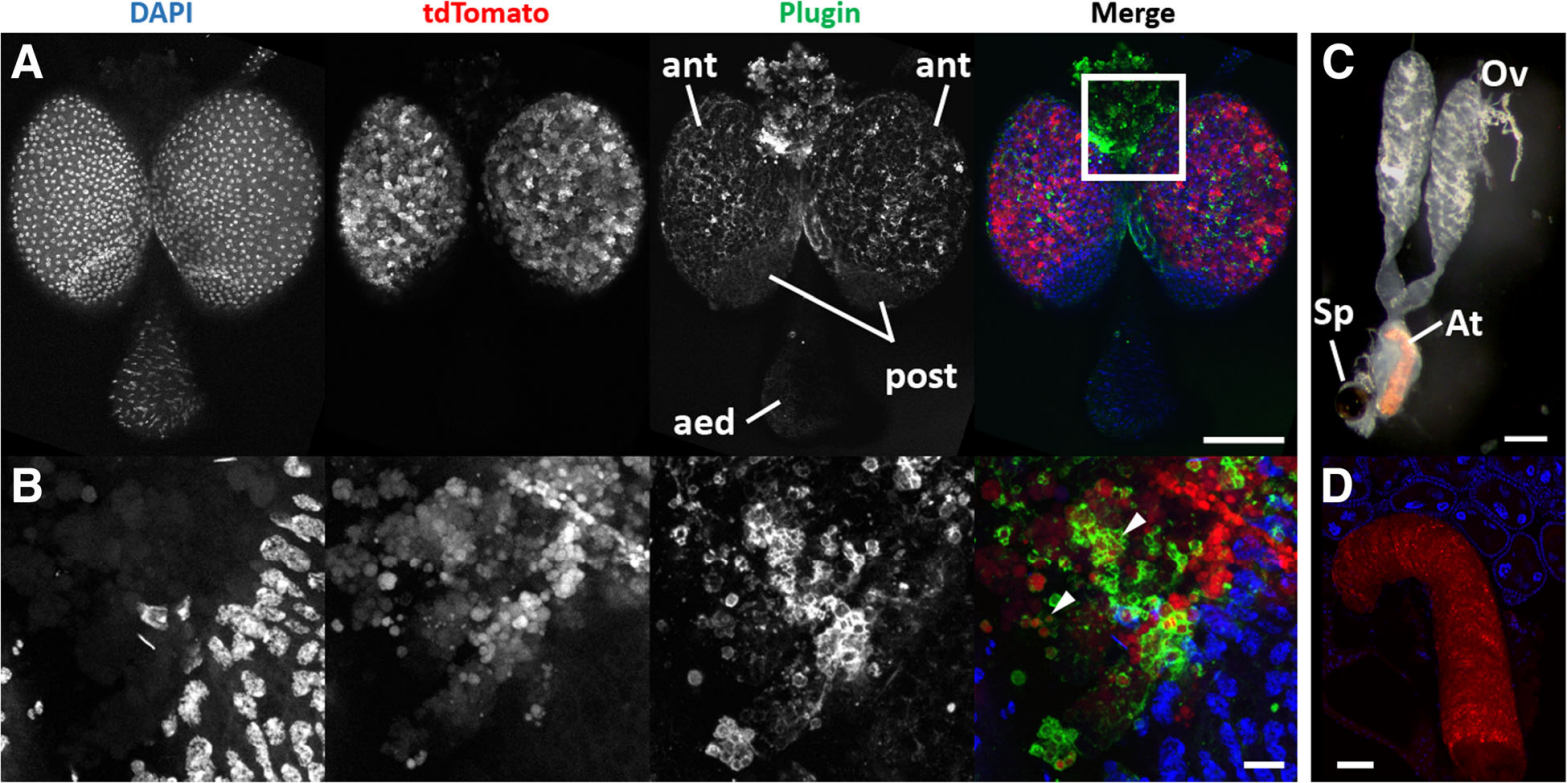
Plugin-tdTomato localization in the MAGs and mating plug. In all panels, Plugin-tdTomato fluorescence is shown in red, Plugin in green, and DNA stained with DAPI in blue. **a** Plugin-tdTomato-filled vesicles occurred throughout the secretory cells of the anterior MAG compartment (ant) but was not detected in the posterior compartment (post) or aedeagus (aed). Stacked Z-projection of the top 3.5 μm. The box in the merge panel represents the region shown in (**b**). **b** Close-up of Plugin and Plugin-tdTomato where MAG epithelial integrity has been disrupted manually. Some Plugin-tdTomato vesicles are co-stained with Plugin (arrowheads). Stacked Z-projection of the top 3.5 μm. **c** The female reproductive tract, consisting of the ovaries (Ov), atrium (At), and the sperm storage organ spermatheca (Sp) immediately after mating. The red fluorescent mating plug is visible within the atrium. **d** A single Z-slice of the mating plug within the female atrium immediately post-mating. Plugin-tdTomato is visible throughout the mating plug, except the tip. *Scale-bars*: (**a**, **c**), 100 μm; (**b**), 20 μm; (**d**), 50 μm

We then performed live fluorescence microscopy and immunofluorescence analysis (IFA) using the α-Plugin antibody on PluTo MAGs. Plugin-tdTomato was localized to vesicles within the anterior MAG compartment (Fig. 3a, b), similar to Plugin in the cryo-EM. Vesicles remained fluorescent as they moved into the interior of the anterior compartment, away from the epithelium. In the IFA, Plugin was localized to channels formed by a muscle network on the outside of the MAGs (Fig. 3a, b), as described previously [20]. It was not detected in vesicles in the interior of the glands, except when MAG epithelium integrity was disrupted (Fig. 3b), suggesting incomplete penetration of the Plugin antibody, and/or the absence of regulatory elements affecting the precise localization pattern of Plugin-tdTomato within the anterior compartment. No Plugin-tdTomato or Plugin staining was detected in the posterior compartment or in the aedeagus.

### Plugin-tdTomato is sexually transferred to the female atrium within the mating plug

To determine if Plugin-tdTomato was incorporated into the plug and transferred to females, we performed forced-mating assays [21, 37] with PluTo males and wild type females, immediately followed by fluorescence imaging. We observed significant tdTomato fluorescence highlighting the characteristic structure of the mating plug within the female atrium (Fig. 3c). Dissection of the female reproductive tract immediately after mating identified a strong fluorescent signal specifically along the length of the mating plug, except the proximal tip, demonstrating that the fusion protein had been incorporated in the plug and transferred during copulation (Fig. 3d).

### Testing PluTo males for *in vivo* assessment of successful mating

**Fig. 4:**
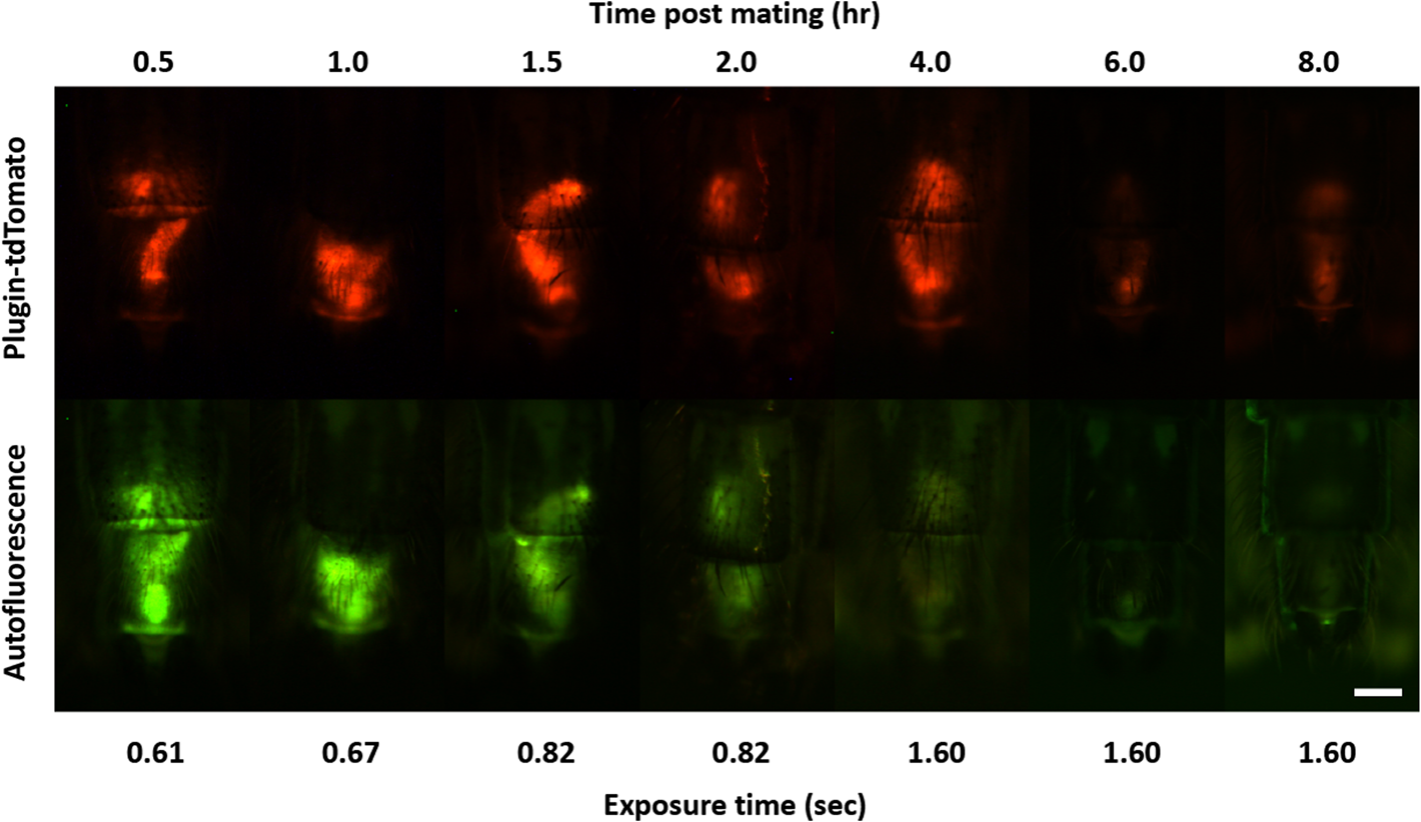
PluTo transgenic males allow *in vivo* assessment of male mating success within the female. Following copulation, the tdTomato-labeled mating plug is distinctly visible through the female cuticle. Autofluorescence is also visible through a wide pass GFP filter set. Mating plug autofluorescence decreases over 4 hours following copulation whereas Plugin-tdTomato fluorescence remains visible for up to 8 hours. Exposure times are indicated below. *Scale-bar*: 100 μm

We next performed a time course analysis to determine whether sexual transfer of a mating plug containing the Plugin-tdTomato fusion could be reliably detected without sacrificing the female. Females mated to PluTo males were analyzed for mating plug fluorescence through the female cuticle at different time points after mating. As previously determined [21], the plug shows naturally-occurring autofluorescence under a filter for green fluorescent protein, but this signal, likely derived from plug lipids, is only reliably detected for 2 hours post mating (Fig. 4). However, in females mated to PluTo males, strong, sharp fluorescence was clearly visible under a red filter for at least 8 hours after mating, demonstrating that this fusion protein can be used to reliably determine the occurrence of successful insemination in a non-invasive manner for a significantly longer span of time (Fig. 4). We did not systematically test later time points as the plug becomes digested and difficult to detect reliably by fluorescence.

### Expression of the Plugin-tdTomato fusion protein does not affect the mating competitiveness of PluTo males and does not impair female reproductive fitness

**Fig. 5:**
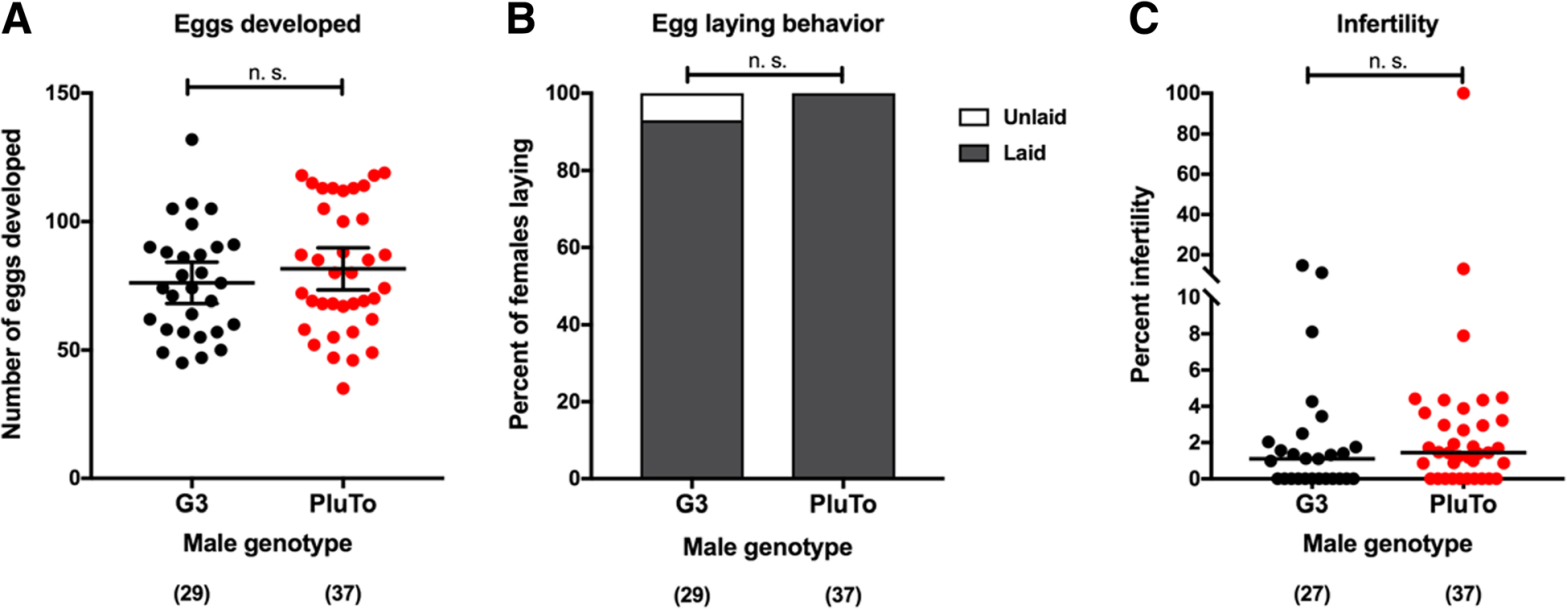
Reproductive fitness of females mated to PluTo males. **a-c** Wild type females mated to either wild type or PluTo males were scored for eggs developed (**a**), egg laying behavior (**b**), and the rate of infertility within broods (**c**). In (**a**) and (**c**), each dot indicates the brood of an individual female. No significant differences were observed between females in the two groups ((**a**): Student’s t-test on square root-transformed data, *P* = 0.38; (**b**): Fisher’s exact test, *P* = 0.189; (**c**): Mann-Whitney U-test, *P* = 0.187)

Given our ability to detect a fluorescent mating plug in mated female for at least 8 hours after copulation, we reasoned that the Plugin-tdTomato fusion protein could be used to assay male mating competitiveness, an important aspect determining the potential success of genetic control programs based on male releases. We therefore assessed whether expression of the transgene affected the mating fitness of PluTo males by performing mating capture assays where 100 PluTo and 100 wild type G3 males were released in cages with 100 wild type virgin females, and mating couples were captured while *in copula*. Fluorescence analysis of 73 couples collected across 4 replicate cages showed no difference in the mating competitiveness of the two groups of males (two-tailed binomial test, *P* = 0.3492), with 44% females inseminated by PluTo individuals. Analysis of the remaining females that were not caught *in copula* but were left in the cage with males confirmed this result, with 33 of 69 mated females (48%) showing the PluTo transgene in sperm stored in their spermathecae (two-tailed binomial test, *P* = 0.810,). Moreover, in separate experiments, females mated to PluTo males had comparable reproductive fitness as females mated to wild type G3 males, measured as the number of eggs developed after blood feeding, and the rates of oviposition and infertility of the broods (Fig. 5a-c). Taken together, these results show that PluTo males induce normal post-mating behavior in females with which they mate, suggesting that Plugin-tdTomato may be a suitable construct for evaluating fitness in mosquito lines for semi-field and field releases.

## Discussion

The control of the *Anopheles* mosquito via the use of LLINs or IRS is key to malaria control strategies and has contributed considerably to the reduction in number of cases and deaths witnessed since the beginning of the century [38]. However, the effectiveness of our best vector control tools is jeopardized by the rapid insurgence and spread of insecticide resistance in *Anopheles* populations, incentivizing the generation of novel methods of genetic control. These methods rely on the release of males that have been modified to express genetic traits that either induce sterility in their female mates, or confer resistance to *Plasmodium* parasites that cause malaria. Some past attempts to release chemical- and radiation-sterilized anophelines reportedly reduced male mating competitiveness [11], which limits the ability of males to sterilize females or to transmit desired genetic traits. While release of sterile males works effectively in other insects [7–9], the intricate mating biology of many anophelines including the major Afrotropical vectors *An. gambiae*, *An. coluzzii*, *An. arabiensis* and *An. funestus* will make developing vector control programs relying on male release difficult in these species. [10, 11, 39, 40]. These species indeed are characterized by copulations in mating swarms, which are formed every night at dusk by large groups of males. Given the highly skewed sex ratio within these swarms, competition between males for the many fewer females is fierce (reviewed in [41]) and a number of factors ranging from the desired genetic manipulations induced in the laboratory to the colonization process and mass rearing, can negatively impact male mating success. Determining the mating competitiveness of males prior to a release is therefore essential for the success of future genetic control efforts.

Currently there are no straightforward methods to determine the mating fitness of released males without sacrificing the female. Available methods necessitate dissections of females shortly after copulation to assess the presence of sperm in the spermatheca and the mating plug in the atrium. While the mating plug is slightly autofluorescent, we show here that this signal can only be reliably detected for a short period of time after copulation. To achieve non-invasive determination of male mating success in competition assays, the only option at present is to blood feed mated females, allow them to oviposit, and screen their progeny for specific genetic traits of the relevant male to determine progeny paternity, while keeping the males alive and in isolation if needed. Our study provides an easy, non-invasive and effective method to assess male mating success in a short period of time and without the need to kill the female. As an example, mating plug-labeled males could be released in a swarm (whether natural or in semi-field cages), and after capturing mating couples, females could be fluorescently analyzed for the occurrence of insemination. Although this method relies on microscopes equipped with fluorescence, which are not likely to be available in remote field settings, the possibility to detect plugs for 8 hours after copulation would allow identification of transgenic matings back in the laboratory. Moreover, although in this study we only tested live females after mating, it is possible that fluorescence will also persist in dead females, such as those captured in light traps, as the fluorescent signal of transgenic lines can generally be observed for a few hours after death.

The *Plugin* upstream regulatory region used in this study was sufficient to achieve MAG-specific expression of our transgene. Such a promoter will facilitate the study of seminal secretions produced by the male glands, for instance via the generation of transgenic RNA interference (RNAi) lines stably expressing double-stranded RNAs (dsRNAs) against targets of interest. Expressing dsRNA transgenes will help to circumvent the limited silencing efficiency achieved by transient injections of dsRNA molecules targeting genes expressed in the MAGs [20, 21], allowing detailed functional analyses.

Finally, by fusing a fluorescent protein to the coding region of Plugin, we ensured incorporation of the marker into the mating plug and its transfer to the female. This system therefore allows for efficient transfer of desired factors from the male to the female, and their slow release once in the female atrium. Interestingly, this property could be utilized to deliver female-specific sterilizing compounds or other factors that may reduce the reproductive fitness of mated females. Although such factors are not readily available, future optimization of the system may afford specific activation of sterilants or toxins only after delivery to the female reproductive tract.

## Conclusions

In conclusion, this study generates a valuable tool for assessing the mating competitiveness of males in semi-field and field studies as well as for laboratory experiments aimed at determining the function of MAG seminal secretions. This tool may prove invaluable when testing the feasibility of releasing genetically modified mosquitoes for the control of malaria-transmitting *Anopheles* populations. Importantly, given the observation that males from the most important Afrotropical anopheline species transfer mating plugs during copulation [23, 42], this system will be readily transferable to other species like *An. arabiensis* and *An. funestus*, key vectors for *Plasmodium falciparum* transmission in sub-Saharan Africa.

## Methods

### Plasmid construction

To clone the PluTo transgene, we separately cloned the promoter, Plugin coding sequence (AGAP009368), and tdTomato with 6S linker into the pDSAY transgenesis vector backbone [35] by standard Golden Gate cloning methods [43]. We amplified 2688 bp of the region upstream of the *Plugin* start codon from genomic DNA isolated from wild type *An. gambiae* (G3 strain) by PCR using GGpP FWD (5'- CAG GTC TCA ATC CTT GTA GGG CTT GTT GAC GGG-3') and GGpP REV (5'- CAGG TCT CAT CAT GTC TAC GGT TGA ATC AGT GAT ACA AGC AAA-3') primers to provide a Golden Gate compatible overhang for the pDSAY destination vector. We amplified the *Plugin* coding sequence from wild type G3 male abdominal cDNA extracts by PCR using GGcP FWD (5'-CCT CTC AAT GAA GGC TTT GGT AGC TCT GCT CTG-3') and GGcP REV (5'-CAG GTC TCA CCT TCG ACG ACC AGC ACA-3') to remove the stop codon, and fuse seamlessly to the *Plugin* promoter fragment. tdTomato was PCR amplified from a plasmid provided by the Tsien Lab [33] using GGtdT FWD (5'-CAG GTC TCA TCC AGC TCC TCC TCC TCC ATG GTG AGC AAG GGC GAG G-3') and GGtdT REV (5'-CAG GTC TCA AAG CTT ACT TGT ACA GCT CGT CCA TGC C-3') primers to add a 6-serine linker and provide a Golden Gate compatible overhang to facilitate cloning into pDSAY. These fragments were cloned into the pDSAY φC31 transgenesis vector backbone [35], which provides an SV40 terminator at the 3' end of Plugin-tdTomato as well as a selectable marker cassette (3xP3-EYFP) to detect transgenic individuals (Fig. 1a).

### Transgenesis and PCR confirmation

Embryo microinjections were performed essentially as described [44, 45] with additional co-injection of a Vasa-φC31 integrase helper plasmid [35] (350 ng/μl and 80 ng/μl of transgenesis and helper plasmids, respectively). A total of 996 X1 docking line embryos were injected, with 172 embryos surviving to hatching. Following outcrossing of the injected P_0_ individuals to wild type G3, a single F_1_ transgenic PluTo male was isolated and outcrossed to G3 females. F_2_ transgenic heterozygotes were intercrossed to yield F_3_ homozygotes. Homozygotes were identified by strong EYFP fluorescence intensity and isolated. Transgene insertion into X1 was confirmed by PCR on three distinct PluTo males and three unintegrated X1 docking line males using the NEB Q5® High-Fidelity 2× Master Mix kit. Whole body mosquitoes were collected and DNA prepared using the Qiagen DNeasy Blood & Tissue Kit. Transgene insertion was confirmed by PCR with EYFP REV (5'-GTC GTC CTT GAA GAA GAT GGT G-3') and X1 FWD (5'- AGG GAA GAT TGG AAT CCA TC-3'). Control PCRs for the empty and unintegrated docking site and S7 controls were performed with X1 FWD (5'-AGG GAA GAT TGG AAT CCA TC-3'), X1 REFV (5'-ACT GCA ACC CAT TCA CAC TG-3'), and S7 FWD (5'-GGC GAT CAT CAT CTA CGT GC-3'), S7 REV (5'-GTA GCT GCT GCA AAC TTC GG-3'), respectively.

### Mosquito maintenance

*Anopheles gambiae* mosquitoes (wild type G3 and PluTo transgenic strains) were reared in cages at 28 °C and 70% humidity on a 12 h/12 h cycle. Adult mosquitoes were fed a 10% (wt/vol) glucose solution *ad libitum* and were given a human blood meal once a week for the purposes of line maintenance. For colony cages, adults were kept in mixed sex cages for up to two weeks post eclosion and one-week post egg-lay. For experiments, males and females were separated as pupae to ensure the virgin status of females and kept in separate cages as experimental conditions demanded. Mating couples were collected either *in copula*, or though forced mating assays (protocol available on https://www.beiresources.org/Publications/MethodsinAnophelesResearch.aspx) and mating was confirmed *via* mating plug autofluorescence or quantitative PCR (below).

### Tissue sample collection for qRT-PCR

Male and female G3 and PluTo mosquitoes were sexed and separated into four cages. From each of these cages, sample groups were sacrificed at 24 h intervals from pupation (d0) until two days post-eclosion (d2). To test whether *Plugin* or *Plugin-tdTomato* expression is constrained to the male accessory glands (MAGs), MAG samples (*n* = 10) were collected from eclosed males and compared to the rest-of-body (ROB) (*n* = 5) while female eclosed mosquitoes were collected as whole body samples (*n* = 5). ROB samples were collected in 200 μl of RNAlater® (Sigma-Aldrich), while MAG samples were transferred to 15 μl of RNAlater using a needle. All samples were stored at -20 °C until specimen collection was completed. Three replicates were performed.

### RNA extraction and cDNA synthesis

ROB samples were defrosted on ice, RNAlater was removed and replaced with 200 μl TRI Reagent® (Applied Biosystems), while 185 μl TRI reagent was added to MAG samples to dilute out the RNA-later and avoid tissue loss. RNA was extracted according to the manufacturer’s instructions. RNA was quantified by using a NanoDrop Spectrophotometer (Thermo Scientific) and DNase treated using Turbo DNAse (Thermo-Fisher). cDNA synthesis was performed as in [46]. Approximately 4 μg of RNA was used in 100 μl cDNA reactions. Reactions were diluted to 200 μl with nuclease-free water for storage at -20 °C.

### Quantitative RT-PCR

Samples for quantitative RT-PCR were diluted tenfold and quantified in triplicate using standard curves. PCRs were run in Fast SYBR Green Master Mix (Thermo-Fisher) on a Step One Plus thermocycler (Applied Biosystems). Endogenous Plugin cDNA was amplified using 9368_FWD (5'-TGA TTC AAC CGT AGA CAT GAA GG-3') and 9368_REV (5'-CCA CCA TAC AAC GGA ACG AC-3') primers. Transgenic Plugin-tdTomato was amplified using qPluginTOM_F (5'-ATC TCA ACA GGA GCC CAA TG-3') and qPluginTOM_R (5'-CCC TTG CTC ACC ATG GAG-3'). Quantities were normalized against the ribosomal protein RpL19 using previously described primers [46].

### Western blotting

Male accessory glands (*n* = 10) and ROB or whole body (*n* = 5) samples from 7-day-old PluTo and G3 age-matched males and females were collected by dissection on ice. Samples were homogenized on ice in protein extraction buffer (25 mM Tris HCl pH 7.4, 150 mM NaCl, 0.1% SDS, 1% Triton X-100, 10 mM EDTA pH 8, 1× protease inhibitor, 1% phosphatase inhibitor) and quantified using a Bradford assay. Protein samples were run at 150 V and 250 mA for 1.5 h on an XCell SureLock vertical system in a 4–12% Bis-Tris NuPAGE gel (Thermo-Fisher). Protein was then transferred to PVDF using iBLOT2 transfer system (Thermo-Fisher). After transfer, membrane was washed twice in 1× PBS-T (0.1% TWEEN in 1× PBS) and blocked in an automated shaker for 1 h at room temperature in Odyssey® Blocking Buffer (PBS) (Li-cor). Membrane was then stained with goat α-tdTomato (Sicgen) and rabbit α-Plugin (GenScript Corp; [20]) (1:1000 concentrations for both antibodies) on an automated shaker at 4 °C overnight. Membrane was washed with PBS-T, and stained with secondary antibodies (donkey α-rabbit 800 and donkey α-goat 680 (Li-cor), both at 1:10,000) in blocking buffer with 0.01% SDS for 1 h at room temperature. Membrane was washed thoroughly before imaging with the Odyssey® CLx imager. Images were analyzed in StudioImageLite (Li-cor).

### Cryo-immune electron microscopy

Virgin 4-day-old male reproductive tracts (including the last three abdominal segments) were fixed overnight at 4 °C (4% paraformaldehyde, 0.1% glutaraldehyde in 0.1 M sodium phosphate buffer at pH 7.4). Samples were washed with 1× PBS, and infiltrated with 2.3 M sucrose in 1× PBS, 0.2 M glycine for 15 min. Tissues were mounted on a pin and frozen in liquid nitrogen prior to sectioning. Samples were sectioned by cryo-Ultra Microtome (Reichert-Jung, Ultracut)(-120 °C) to obtain thick sections (0.5 μm) for visual screening with toluidine blue, or ultrathin sections (70–80 nm) for target tissue analysis. Ultrathin sections were transferred to carbon-coated copper grids, blocked in 1× PBS, 1% BSA for 10 min and stained 30 min with primary antibody at RT (1:30 dilutions of rabbit α-Plugin). The grids were washed in 1× PBS for 15 min and then labelled with Protein-A gold (Sigma-Aldrich, 15 nm diameter) diluted in blocking solution for 20 min, following a final wash in distilled water for 15 min. Contrasting of the labelled grids was carried out on ice in 0.3% uranyl acetate in 2% methyl cellulose for 10 min. All micrographs were captured using a JEOL 1200EX 80 kV electron microscope and recorded with an AMT 2k CCD camera. Three biological replicates were performed, and a representative selection are shown in the text. Samples were prepared by the Harvard Medical School EM core facility.

### Fluorescence imaging

MAGs were collected from 5–7 day-old virgin PluTo males and fixed in 4% paraformaldehyde solution for 1 h, then rinsed 1× PBS. Tissues were soaked in 80% ethanol at 4 °C for 3 min to remove lipids, rinsed 3 times in 1× PBS, bleached in 3% hydrogen peroxide for 5 min to quench endogenous autofluorescence, and rinsed in 1× PBS 3 times. Samples were blocked and permeabilized overnight in blocking solution (1% BSA, 0.3% Tween-20 in 1× PBS), then stained with rabbit α-Plugin (1:200) for approximately 12 h. Tissues were washed 5 times in 1× PBS followed by a 30 min incubation in blocking solution. Tissues were then stained for 1 h at room temperature with secondary antibody (Alexafluor 647 donkey anti-rabbit (1:1000) in blocking solution, rinsed in blocking solution, then stained with DAPI (5 μg/ml) in blocking solution for 10 min. Tissues were washed at room temperature 5 times with blocking solution, once with PBS-T, and twice with 1× PBS. Tissues were then mounted in Vectashield® Mounting Medium (Vector Laboratories) and imaged using an upright SPE confocal microscope (Harvard NeuroDiscovery Center Leica SPE). Live unfixed samples were dissected on ice in 1× PBS and imaged immediately on a Leica dissecting microscope with fluorescence.

### Mating plug digestion and reproductive fitness assays

Four-day-old virgin G3 females were forced mated to four-day-old PluTo or G3 males and screened for mating plugs. Females without a visible mating plug were excluded from the study. Females force mated to PluTo males were then further divided into 4 groups of 10 individuals and each group imaged under fluorescent lighting at t = 0.5, 1, 1.5, 2, 4, 6 and 8 h post-mating. After imaging, females were blood-fed, placed in isolated oviposition cups and given 4 d to lay eggs, after which total numbers of eggs were tallied and infertility was scored.

### Mating competitiveness assay

Reproductive behavioral effects on male mating efficacy resulting from our transgene were evaluated using a mating competitiveness assay where 100 G3 and 100 PluTo males (4–5-day-old) were allowed to compete and mate freely for 100 G3 females (4-day-old) in an open cage environment. Couples were caught *in copula* for 1 h as previously described [46], and male genotype determined by fluorescence of the PluTo transgene. Uncaptured females were left in the cage for 24 h, after which females were collected and individually processed for DNA by brief homogenization in extraction buffer (0.12% Tris-Cl, 0.037% EDTA, 0.29% NaCl). Mating status and male genotype were determined using a modified qPCR protocol using both Y-specific primers YQPCR_FWD (5'-GGA TCT GGC CAA GAG GAG TA-3'), YQPCR_REV (5'-CCC AAC CAA GGT ACT CTA ACG-3'), and tdTomato primers Q485 (5'-TGG AGT TCA AGA CCA TCT AC-3'), Q486 (5'-GTG TCC ACG TAG TAG TAG CC-3').

### Statistical analysis

Data were analyzed using GraphPad Prism 6.0, with statistical tests used indicated in the figure legends. Quantitative RT-PCR: since expression of Plugin-tdTomato was not detectable in the G3 background, precluding a full multi-factorial analysis (genotype × gene × time), we analyzed Plugin and Plugin-tdTomato expression levels using two-way analysis of variance incorporating age (0, 1, and 2 d) and genotype-primer combination (G3-Plugin, PluTo-Plugin, PluTo-Plugin-tdTomato) as factors, followed by Tukey’s multiple comparison test, with adjusted p-values for multiple testing.

## Additional files

Additional file 1: **Figure S1**. Verification of PluTo transgene insertion in the X1 docking site. Three PluTo and X1 adult males were genotyped for the presence of the PluTo transgene inserted within the docking site, the empty docking site, or the ribosomal gene RpS7 as DNA quality control. PCR primers specific to the PluTo transgene as well as the X1 docking site sequence were used to amplify the transgene specifically within the site. For comparison, the empty X1 docking site, with an untransformed attP sequence was also verified in X1 individuals. (TIF 101 kb)

Additional file 2: **Figure S2**. Endogenous Plugin and PluTo transgene expression. **A** Quantitative qRT-PCR of endogenous *Plugin* and *Plugin-tdTomato* expression levels in male and female PluTo and G3 pupae and adult tissues over time. An age of zero days refers to uneclosed pupae (not dissected) and ages 1 and 2 are minimal ages in days post-eclosion (MAGs were dissected out of males). In MAGs, transgenic *Plugin-tdTomato* was induced similarly to endogenous *Plugin* levels, and age significantly affected expression. In male rest of body tissues, transgenic *Plugin-tdTomato* was expressed at negligible, although higher, levels at ages 1 and 2 days. In female whole body tissues, transgenic *Plugin-tdTomato* was also expressed at negligible levels. Endogenous *Plugin* levels could not be reliably detected in wild type females aged 1 or 2 days. Analysis of variance followed by post-hoc testing within ages showed the following significant differences: MAGs (left panel): ANOVA (Age *F*_(2, 15)_ = 16.1, *P* = 0.0002, Genotype-Primer combination *F*_(2, 15)_ = 2.7, *P* = 0.099, Interaction *F*_(4, 15)_ = 1.92, *P* = 0.160). Tukey’s post-hoc test: Age 1 - PluTo *Plugin-tdTomato vs* G3 *Plugin* (mean difference ± SEM = 43.3 ± 16.63), adj. *P* value = 0.0359. Male Rest of body (center panel) ANOVA (Genotype-Primer combination *F*_(2, 11)_ = 12.3, *P* = 0.0015, Age *F*_(1, 11)_ = 1.99, *P* = 0.186, Interaction *F*_(2, 11)_ = 0.53, *P* = 0.601). Tukey’s post-hoc tests: Age 1 - PluTo *Plugin-tdTomato vs* PluTo *Plugin* (mean difference ± SEM = 0.021 ± 0.005), adj. *P* value = 0.0067; Age 2 - PluTo *Plugin-tdTomato vs* PluTo *Plugin* (mean difference ± SEM = 0.015 ± 0.005), adj. *P* value = 0.0406; Age 2 - PluTo *Plugin-tdTomato vs* G3 *Plugin* (mean difference ± SEM = 0.015 ± 0.005), adj. *P* value = 0.037. The downward error bar for Age 1 G3 *Plugin* cannot be plotted on a logarithmic axis as it extends below 0. Female Rest of body (right panel): ANOVA (Genotype-Primer combination *F*_(2, 16)_ = 4.88, *P* = 0.022, Age *F*_(2, 16)_ = 0.638, *P* = 0.541, Interaction *F*_(4, 16)_ = 0.6, *P* = 0.668). Tukey’s post-hoc tests: no pairwise comparisons were detected with adj. *P* value < 0.05. **B** Western blot of endogenous Plugin and Plugin-tdTomato in adult male and female tissues with α-Plugin in green, and α-tdTomato in red. Endogenous Plugin is observed at similar levels in the MAGs of both wild type G3 and PluTo transgenics at ~80 kDA (band 2). The α-Plugin antibody also recognizes an additional band at 27 kDA (band 1), which may be non-specific or cleaved protein. Full length transgenic Plugin-tdTomato is observed at ~150 kDA recognized by both α-Plugin antibody and α-tdTomato (band 4). An additional Plugin-tdTomato species at 120 kDA is recognized exclusively by the α-tdTomato antibody (band 3), which may represent cleaved protein, or result from an alternative start codon excluding the α-Plugin antibody binding site. Multiple high molecular weight bands (bands 6) are observed as dimers and multimers of Plugin and Plugin-tdTomato species in wild type G3 and PluTo males, respectively. Detectable Plugin and Plugin-tdTomato protein are not observed in male rest-of-body nor female whole-body tissues. The α-tdTomato antibody recognizes unspecific low molecular weight moieties in these tissues (bands 5) (TIF 281 kb)

## Abbreviations

G3: Wild type strain of *An. gambiae* mosquitoes
IRS: Indoor residual sprays
LLIN: Long-lasting insecticide treated nets
LRT: Lower reproductive tract (female)
MAGs: Male accessory glands
PluTo: Transgenic *An. gambiae* line expressing Plugin-tdTomato in the MAGs
X1: A φC31-based transgenic docking line
SEM: Standard error of the mean
