# Supplementary figures and images for: A transgenic tool to assess Anopheles mating competitiveness in the field
Source: Parasit Vectors. 2018 Dec 24;11(Suppl 2):651. doi: 10.1186/s13071-018-3218-5 (PMC6304768; doi:10.1186/s13071-018-3218-5)

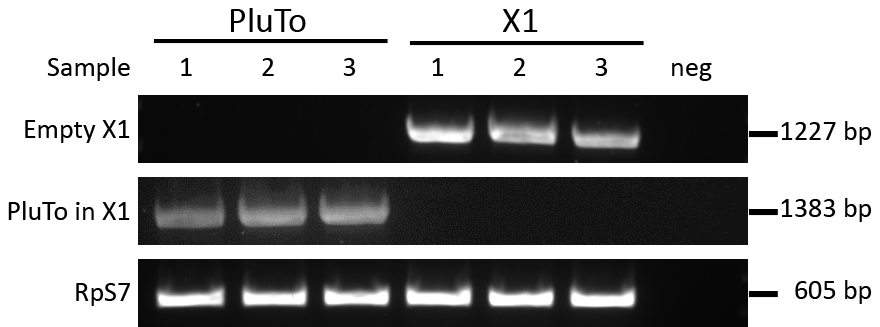

Supplement: Supplementary file 1 — Figure S1. Verification of PluTo transgene insertion in the X1 docking site. Three PluTo and X1 adult males were genotyped for the presence of the PluTo transgene inserted within the docking site, the empty docking site, or the ribosomal gene RpS7 as DNA quality control. PCR primers specific to the PluTo transgene as well as the X1 docking site sequence were used to amplify the transgene specifically within the site. For comparison, the empty X1 docking site, with an untransformed attP sequence was also verified in X1 individuals. (TIF 101 kb) [file 13071_2018_3218_MOESM1_ESM.tif]

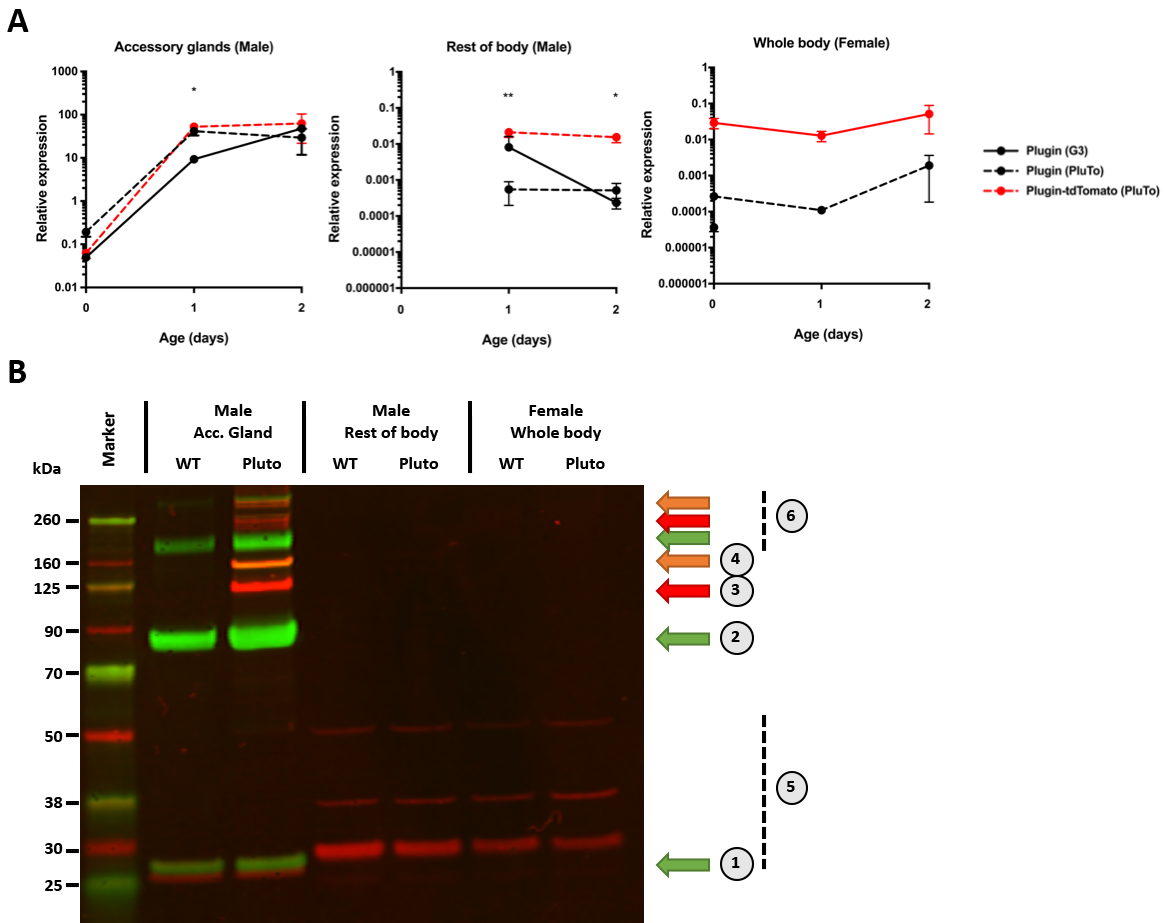

Supplement: Supplementary file 2 — Figure S2. Endogenous Plugin and PluTo transgene expression. A Quantitative qRT-PCR of endogenous Plugin and Plugin-tdTomato expression levels in male and female PluTo and G3 pupae and adult tissues over time. An age of zero days refers to uneclosed pupae (not dissected) and ages 1 and 2 are minimal ages in days post-eclosion (MAGs were dissected out of males). In MAGs, transgenic Plugin-tdTomato was induced similarly to endogenous Plugin levels, and age significantly affected expression. In male rest of body tissues, transgenic Plugin-tdTomato was expressed at negligible, although higher, levels at ages 1 and 2 days. In female whole body tissues, transgenic Plugin-tdTomato was also expressed at negligible levels. Endogenous Plugin levels could not be reliably detected in wild type females aged 1 or 2 days. Analysis of variance followed by post-hoc testing within ages showed the following significant differences: MAGs (left panel): ANOVA (Age F(2, 15) = 16.1, P = 0.0002, Genotype-Primer combination F(2, 15) = 2.7, P = 0.099, Interaction F(4, 15) = 1.92, P = 0.160). Tukey’s post-hoc test: Age 1 - PluTo Plugin-tdTomato vs G3 Plugin (mean difference ± SEM = 43.3 ± 16.63), adj. P value = 0.0359. Male Rest of body (center panel) ANOVA (Genotype-Primer combination F(2, 11) = 12.3, P = 0.0015, Age F(1, 11) = 1.99, P = 0.186, Interaction F(2, 11) = 0.53, P = 0.601). Tukey’s post-hoc tests: Age 1 - PluTo Plugin-tdTomato vs PluTo Plugin (mean difference ± SEM = 0.021 ± 0.005), adj. P value = 0.0067; Age 2 - PluTo Plugin-tdTomato vs PluTo Plugin (mean difference ± SEM = 0.015 ± 0.005), adj. P value = 0.0406; Age 2 - PluTo Plugin-tdTomato vs G3 Plugin (mean difference ± SEM = 0.015 ± 0.005), adj. P value = 0.037. The downward error bar for Age 1 G3 Plugin cannot be plotted on a logarithmic axis as it extends below 0. Female Rest of body (right panel): ANOVA (Genotype-Primer combination F(2, 16) = 4.88, P = 0.022, Age F(2, 16) = 0.638, P = 0.541, Interaction F(4, 16) = 0.6, P = [file 13071_2018_3218_MOESM2_ESM.tif]
